# Comparative changes in cardiovascular measures following speed, agility, and quickness training and small-sided games in youth football players: a randomized trial

**DOI:** 10.1186/s13102-026-02048-2

**Published:** 2026-09-10

**Authors:** Mahmoud Al Sayed, Mohamed Meselhy, Mohamed Bakr

**Affiliations:** 1 Department of Health Sciences Sports, Faculty of Sport Science, Benha University, Benha, Egypt; 2 Department of Theories and Application of Team Sports and Racket Sports, Faculty of Sport Science, Benha University, Benha, Egypt

**Keywords:** Youth football, Small-sided games, Speed, agility, and quickness training, Cardiovascular measures, Exercise intervention, Randomized trial

## Abstract

**Backgroun:**

Speed, Agility, and Quickness (SAQ) training and small-sided games (SSGs) are widely used in youth football but impose different physical and football-specific demands. Evidence directly comparing their cardiovascular responses following a structured training intervention remains limited.

**Objective:**

To compare cardiovascular response patterns following an 8-week SAQ or SSG training programme in male youth football players.

**Methods:**

Thirty-six male youth football players were randomly allocated to an SAQ group (*n* = 18) or an SSG group (*n* = 18). Cardiovascular outcomes were assessed before and after the 8-week intervention and included resting heart rate (HR), post-shuttle-run HR, systolic blood pressure (SBP), and diastolic blood pressure (DBP). Pre-to-post changes were examined using a two-way mixed-design analysis of variance, with time as the within-subject factor and group as the between-subject factor.

**Results:**

Both groups demonstrated reductions in all four cardiovascular outcomes. Significant time × group interactions were observed for resting HR, post-shuttle-run HR, SBP, and DBP. SSG training produced greater numerical reductions in resting HR, SBP, and DBP, whereas SAQ training produced a slightly greater reduction in post-shuttle-run HR.

**Conclusion:**

The findings indicate different cardiovascular response patterns following SAQ and SSG training rather than universal superiority of either modality. SSGs may be useful when cardiovascular conditioning is integrated with football-specific technical and tactical demands, whereas SAQ training provides a more targeted stimulus for acceleration, agility, and neuromuscular performance.

**Trial registration:**

ClinicalTrials.gov NCT07633210, registered on 03/06/2026. Retrospectively registered.

**Supplementary Information:**

The online version contains supplementary material available at https://doi.org/10.1186/s13102-026-02048-2.

## Introduction

Football is an intermittent team sport characterized by frequent changes in movement intensity, including walking, jogging, acceleration, deceleration, sprinting, and rapid changes of direction, combined with continuous technical and tactical interactions [1–3]. These demands are particularly relevant during youth development, when physical conditioning must be integrated with the progressive acquisition of football-specific technical and tactical skills. Consequently, training programmes for young football players commonly combine general conditioning methods with sport-specific activities designed to reproduce relevant physiological and movement demands [3, 4]. Among the conditioning strategies frequently used in football are Speed, Agility, and Quickness (SAQ) training and small-sided games (SSGs), although these approaches differ substantially in their structure, movement demands, and degree of football specificity [5–7].

SAQ training comprises exercises targeting acceleration, rapid changes of direction, movement speed, and quick responses to external stimuli. Its structured nature allows coaches to manipulate running distance, movement direction, repetitions, recovery intervals, and exercise complexity while emphasizing specific movement qualities. Previous research in youth football has reported improvements in sprinting, agility, and change-of-direction performance following SAQ-based interventions [6, 8, 9]. For example, Trecroci et al. reported improvements in selected physical and cognitive outcomes following an SAQ programme in preadolescent soccer players [6]. More recently, a systematic review and meta-analysis reported beneficial effects of SAQ training on several physical-performance outcomes, including sprinting, change-of-direction ability, power, and change-of-direction dribbling [8]. However, considerable heterogeneity exists among SAQ interventions with respect to exercise selection, training frequency, duration, intensity, and participant characteristics [8]. Thus, although evidence supporting SAQ for physical performance is increasing, its effects on cardiovascular responses in youth football players remain less clearly established.

In contrast, SSGs provide a football-specific conditioning strategy in which physical activity is performed concurrently with technical, tactical, perceptual, and decision-making demands. By manipulating player numbers, pitch dimensions, playing rules, team organization, and task constraints, coaches can substantially modify the physiological, physical, technical, and perceptual demands imposed on players [7, 10–20]. Previous research has demonstrated that changes in game format, relative playing area, player number, and rule constraints can alter heart-rate responses, running demands, perceived exertion, and other physiological and activity-related outcomes [10–20]. In youth players, these task constraints can produce meaningful differences in physiological and physical responses across SSG configurations [15, 16]. SSGs therefore provide an ecologically valid training environment in which cardiovascular loading occurs simultaneously with passing, receiving, dribbling, defending, opponent interactions, and tactical decision-making [7, 10–13, 21].

The physiological stimulus generated by SSGs is consequently not uniform. Differences in pitch dimensions, player numbers, game format, rules, and tactical constraints can alter both the intensity and nature of the training stimulus [13–20]. Such findings emphasize that the cardiovascular demands of an SSG intervention cannot be interpreted independently of its specific training structure. This consideration is particularly important when SSGs are compared with more structured conditioning methods because differences in cardiovascular responses may reflect not only exercise intensity but also the way in which the training stimulus is generated.

The distinction between SAQ and SSG training is therefore both theoretically and practically important because these modalities are not interchangeable conditioning methods and emphasize different components of athletic development. SAQ training provides a relatively controlled neuromuscular stimulus, allowing repeated exposure to acceleration, rapid footwork, deceleration, and change-of-direction actions under predetermined work and recovery conditions [6, 8, 22]. Its primary emphasis is on movement quality, acceleration, rapid movement execution, and change-of-direction performance. In contrast, SSGs provide an integrated football-specific stimulus in which locomotor and cardiovascular demands occur concurrently with technical execution, tactical interaction, opponent pressure, perception, and decision-making [7, 10–18, 21]. Comparative research in young soccer players has demonstrated that SAQ and SSG training can produce different physical-performance adaptations [22], supporting the view that these approaches should be considered distinct training strategies rather than equivalent forms of conditioning.

Previous research comparing structured conditioning activities with game-based training further supports this distinction. Structured drills permit greater control over movement direction, repetition number, work duration, recovery interval, and exercise complexity, thereby allowing coaches to emphasize specific neuromuscular qualities [6, 8, 22]. Game-based training, in contrast, exposes players to continuously changing locomotor, technical, tactical, perceptual, and decision-making demands [7, 10–18, 21]. Although both approaches can generate substantial cardiovascular loading, the internal and external demands through which this loading is produced may differ. SAQ training emphasizes repeated high-quality acceleration, rapid movement, and change-of-direction actions, whereas SSGs integrate locomotor and cardiovascular activity with technical execution, tactical interaction, and perceptual decision-making [6, 7, 10–13, 21, 22]. These differences provide a rationale for directly comparing cardiovascular responses associated with SAQ and SSG training rather than assuming that the two modalities provide equivalent conditioning stimuli.

The potential value of SSG training extends beyond its immediate physiological demands. Meta-analytic research indicates that SSGs can provide a viable alternative or complement to conventional endurance and running-based high-intensity training for developing physical and endurance-related performance in soccer players [23–25]. However, the magnitude and nature of these responses depend on characteristics of the SSG programme, including training frequency, duration, player number, pitch dimensions, relative playing area, and task constraints [14–20, 23–25]. Consequently, findings from one SSG protocol cannot necessarily be generalized to other SSG programmes. The specific organization of the intervention must therefore be considered when interpreting cardiovascular outcomes following SSG training.

Heart rate and blood pressure are practical cardiovascular outcomes for field-based sports research because they can be assessed repeatedly using standardized procedures. Heart rate provides an indicator of internal physiological load and reflects the cardiovascular response to the intensity and duration of an exercise task [26, 27]. Resting heart rate and blood pressure provide complementary information about cardiovascular status, whereas heart rate measured immediately following a standardized exercise challenge provides information about the response to a controlled external stimulus. However, cardiovascular response should not be equated directly with long-term cardiovascular fitness. A higher heart rate during exercise indicates greater physiological demand under the conditions of that exercise bout but does not necessarily indicate greater long-term adaptation or superior overall training effectiveness. Similarly, post-exercise heart rate alone should not be interpreted as direct evidence of changes in aerobic fitness. Direct measures such as VO₂max or VO₂peak would provide stronger evidence of cardiorespiratory fitness adaptations.

The 20-m multistage shuttle-run test (20-mSRT) is a widely used field-based assessment involving repeated 20-m runs at progressively increasing speeds controlled by standardized auditory signals. The original protocol was developed as a practical field test for estimating aerobic fitness across different populations [28]. Although the shuttle-run test can provide an indirect estimate of aerobic capacity, the present study used heart rate immediately following the standardized shuttle-run test as an exercise-related cardiovascular outcome. Accordingly, post-test heart rate was interpreted as the cardiovascular response to a standardized exercise challenge rather than as a direct measure of VO₂max or definitive evidence of changes in cardiorespiratory fitness.

Another important consideration is that cardiovascular responses may differ according to the characteristics of the training stimulus. SSGs can generate substantial cardiovascular demands because players repeatedly perform football-specific movements while responding to technical and tactical constraints [10, 12–20]. In contrast, SAQ exercises generally consist of short, high-quality bouts of acceleration, rapid movement, and change of direction interspersed with recovery periods. These differences in exercise structure may influence the cardiovascular stimulus delivered during training and the subsequent pattern of pre-to-post response. However, cardiovascular responses cannot be interpreted independently of other components of training load. Measures such as rating of perceived exertion, blood lactate, and GPS-derived external load can provide complementary information about internal and external demands [27], while technical, tactical, perceptual, and neuromuscular measures can help characterize the broader training stimulus. A multidimensional assessment is therefore important when determining the overall effects of different football-conditioning strategies.

This distinction is also relevant from a practical coaching perspective. SAQ training may be particularly useful when the objective is to emphasize acceleration, agility, rapid movement, and neuromuscular qualities, whereas SSGs may be advantageous when conditioning is intended to occur concurrently with football-specific technical, tactical, perceptual, and decision-making activity [6–8, 22]. In practice, these approaches are not necessarily mutually exclusive and may be integrated within periodized training programmes according to the objectives of a particular session or training phase. Comparing their cardiovascular response patterns may therefore help coaches select and combine conditioning strategies according to specific developmental objectives rather than assuming that one modality is universally superior.

Despite increasing evidence concerning both SAQ and SSG training, relatively little research has directly compared their cardiovascular response patterns following a structured intervention in youth football players. Existing studies have predominantly focused on physical-performance outcomes following SAQ or comparative interventions [6, 8, 22], or on acute physiological, locomotor, perceptual, and technical responses during specific SSG formats [7, 10–21]. Although comparative studies have demonstrated that SAQ and SSG training may produce different physical-performance responses [22], evidence concerning whether these modalities produce different changes in resting and exercise-related cardiovascular measures following a controlled training intervention remains limited. Furthermore, evidence from individual SSG configurations cannot necessarily be generalized to all SSG programmes because cardiovascular demands are strongly influenced by game format, playing area, player number, and task constraints [13–20]. A direct randomized comparison of standardized SAQ and SSG programmes may therefore provide useful evidence concerning their different cardiovascular response patterns in youth football players.

Therefore, the purpose of this randomized controlled trial was to compare changes in selected cardiovascular measures following an 8-week SAQ training programme and an SSG programme in male youth football players. The primary outcomes were resting heart rate, heart rate immediately following a standardized 20-m shuttle-run test, systolic blood pressure, and diastolic blood pressure. We hypothesized that the magnitude and pattern of pre-to-post changes in these cardiovascular measures would differ between the SAQ and SSG groups because of differences in their physiological, neuromuscular, technical, perceptual, and football-specific training demands. The study was designed to compare two active conditioning modalities and was not intended to establish that either modality was universally superior or that observed pre-to-post changes were attributable exclusively to the intervention independently of normal development, maturation, habitual training, or other contextual factors.

## Methods

### Study design

A controlled, parallel-group intervention design was used to compare cardiovascular adaptations following two football-specific training modalities: speed, agility, and quickness (SAQ) training and small-sided games (SSGs). The intervention lasted 8 consecutive weeks, with three supervised training sessions per week, resulting in 24 training sessions for each group.

Participants were allocated in a 1:1 ratio to either the SAQ training group or the SSG training group. Baseline cardiovascular assessments were conducted before commencement of the intervention, and the same assessment procedures were repeated following completion of the 8-week training programme.

The study was designed to examine comparative adaptations between two active training modalities. Because both groups received structured experimental training without a passive or usual-training comparison group, changes from baseline to post-intervention were interpreted within the context of the respective training modality. The principal comparison was the difference in pre-to-post change between the SAQ and SSG groups. Accordingly, the findings were interpreted as comparative responses to two active training conditions rather than as evidence that either intervention produced changes relative to normal development, maturation, habitual training, or the absence of additional training.

All participants continued their usual football-related activities outside the experimental sessions. The experimental sessions were incorporated into their regular football-training schedule. Both groups completed an identical standardized warm-up and cool-down, whereas the experimental training component differed according to group allocation.

The study was reported in accordance with applicable principles of the Consolidated Standards of Reporting Trials (CONSORT) framework.

**Fig. 1:**
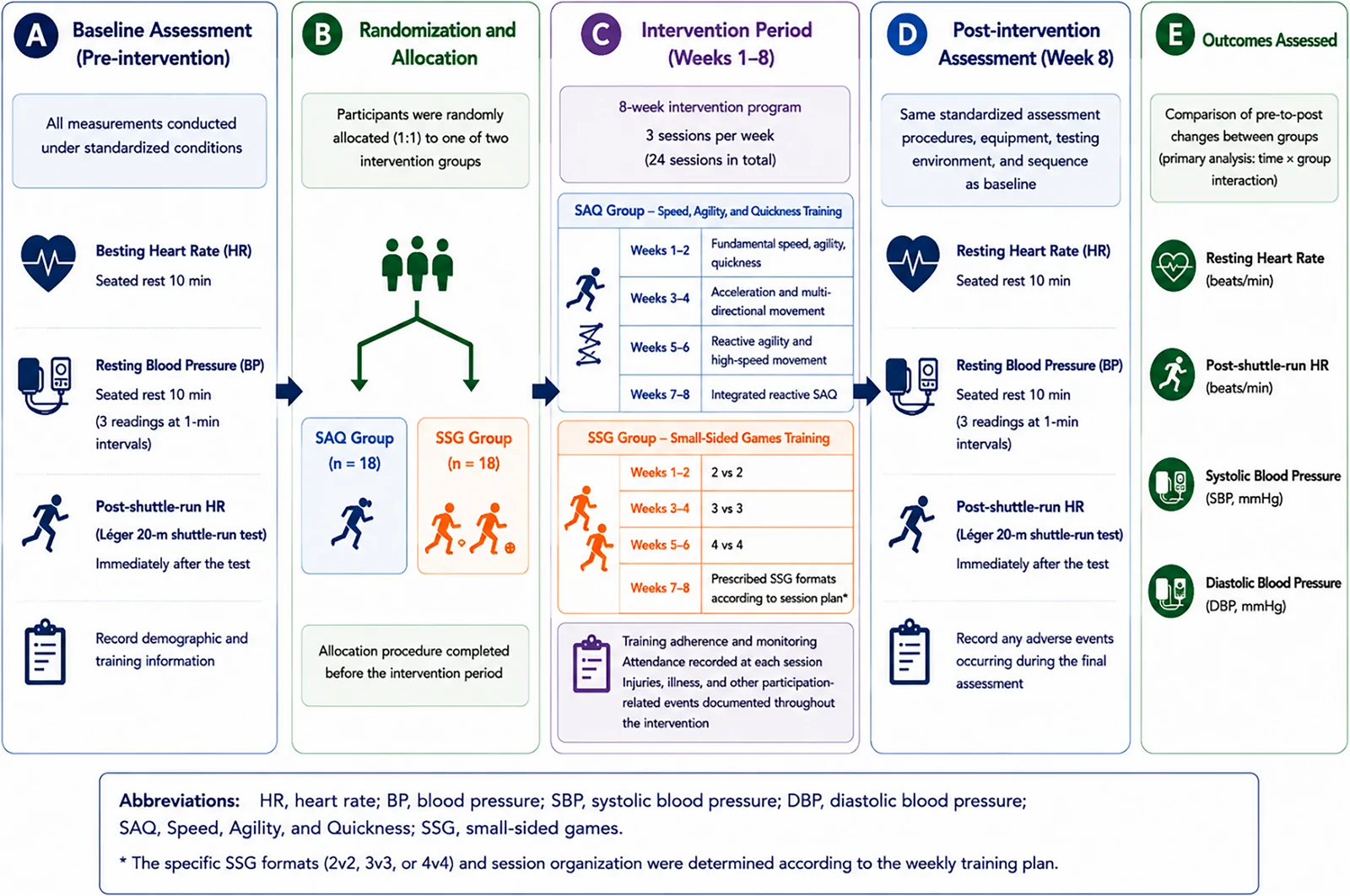
Experimental design and data-collection timeline

The primary cardiovascular outcomes were resting heart rate (HR), post-shuttle-run HR, systolic blood pressure (SBP), and diastolic blood pressure (DBP). An overview of participant allocation, intervention duration, training frequency, and the timing of cardiovascular assessments is presented in Fig. 1.

### Participants

Thirty-six male youth football players participated in the study. Participants were allocated equally between the two intervention groups, with 18 participants in the SAQ group and 18 participants in the SSG group.

Participants were recruited from a competitive football-training environment and were regularly engaged in organized football practice. Eligibility required participants to be physically capable of completing the prescribed training programme and cardiovascular testing procedures.

Participants were excluded if they had a current musculoskeletal injury, acute illness, known cardiovascular condition, or other medical condition that prevented participation in vigorous football training or exercise testing.

Before participation, the study procedures, potential risks, and potential benefits were explained to the participants and their parents or legal guardians. Written informed consent was obtained from the parents or legal guardians, and participant assent was obtained where applicable.

Participants were instructed to maintain their usual dietary and lifestyle habits throughout the intervention and to avoid additional structured high-intensity training that could substantially alter the experimental training stimulus.

All participants included in the final analysis completed both baseline and post-intervention assessments.

### Biological maturation

Biological maturation was not directly assessed using skeletal age, Tanner staging, peak height velocity (PHV), maturity offset, or another maturity-specific assessment. Participants were recruited from the same organized youth football environment and underwent the same eligibility procedures, intervention schedule, and cardiovascular assessment protocols.

Because biological maturation can influence cardiovascular function, physical capacity, body size, and responsiveness to training during adolescence, maturational variability was considered when interpreting the comparative cardiovascular responses between the SAQ and SSG groups.

### Randomization, allocation concealment, and outcome assessment

Following eligibility screening and completion of the baseline assessments, participants were randomly allocated in a 1:1 ratio to either the Speed, Agility, and Quickness (SAQ) training group or the Small-Sided Games (SSG) training group, with 18 participants assigned to each group. Group allocation was completed before commencement of the 8-week intervention.

The study records confirm that participants were randomly allocated to the two intervention groups; however, the specific sequence-generation procedure and the method used to conceal group allocation at the time of assignment were not documented in sufficient detail to permit reconstruction of the allocation process. Accordingly, no specific allocation-concealment method, such as sealed opaque envelopes, centralized allocation, or a computer-based allocation service, is reported.

Outcome assessments were conducted using standardized procedures and the same measurement equipment at baseline and post-intervention. The available records do not permit confirmation that outcome assessors were formally blinded to group allocation. Therefore, assessor blinding was not assumed. To minimize potential assessment bias, cardiovascular outcomes were measured using objective physiological instruments and predefined measurement procedures, with the same testing sequence, equipment, rest periods, and measurement procedures applied at both assessment points.

### Training intervention

The intervention was conducted over 8 consecutive weeks, with three training sessions per week, resulting in 24 supervised training sessions per group.

Training sessions were distributed across the weekly training schedule to provide adequate recovery between experimental sessions. Whenever possible, sessions were conducted at approximately the same time of day and under comparable environmental and facility conditions.

Each intervention session consisted of:

1. A standardized 10-min warm-up;
2. The group-specific experimental training component (SAQ or SSG); and
3. A standardized 5-min cool-down.

The experimental component was exclusive to the participant’s assigned group. Participants in the SAQ group completed the prescribed SAQ programme, whereas participants in the SSG group completed the prescribed SSG programme.

Training volume and intensity were progressively structured throughout the 8-week intervention. In the SAQ group, progression was achieved through manipulation of repetitions, sets, work duration, recovery intervals, and movement complexity. In the SSG group, progression was achieved through changes in player number, playing area, bout duration, and associated technical, perceptual, spatial, and tactical demands.

The detailed SAQ and SSG prescriptions are presented in Tables 1, 2, 3 and 4.

**Table 1:** SAQ training prescription across the 8-week intervention

Week	Training emphasis	Sets	Repetitions	Work duration	Recovery between repetitions	Recovery between sets	Approximate SAQ duration
1	Fundamental speed, agility, and quickness	2	4	10 s	40 s	90 s	12–15 min
2	Acceleration and planned agility	2	5	10 s	40 s	90 s	13–16 min
3	Acceleration and multidirectional agility	3	4	10–12 s	40 s	90 s	15–17 min
4	Increased volume and movement complexity	3	5	10–12 s	35 s	90 s	16–18 min
5	High-speed multidirectional movement	3	5	12 s	35 s	75 s	17–19 min
6	Increased repetition volume	3	6	12 s	35 s	75 s	18–20 min
7	High-intensity SAQ	3	6	12–15 s	30 s	60 s	19–21 min
8	High-intensity football-specific SAQ	3	6	15 s	30 s	60 s	20–22 min

Work duration refers to the active execution period of each drill. Recovery between repetitions refers to the interval between successive repetitions within a set, whereas recovery between sets refers to the interval separating consecutive sets. The work-to-recovery ratio progressed from approximately 1:4 during Weeks 1–2 to approximately 1:2 during Week 8, reflecting a progressive increase in training density. Individual drill duration could vary slightly according to the specific exercise, but the prescribed weekly sets, repetitions, work intervals, and recovery periods were maintained

*SAQ* Speed, agility, and quickness

**Table 2:** Standardized characteristics of the SSG formats

SSG format	Players	Pitch dimensions	Pitch area	Area per player	Bout duration	Bouts/session	Recovery between bouts	Total active SSG time
2 vs. 2	4	12 × 24 m	288 m²	72 m²/player	2 min	8	2 min	16 min
3 vs. 3	6	18 × 30 m	540 m²	90 m²/player	3 min	8	2 min	24 min
4 vs. 4	8	24 × 36 m	864 m²	108 m²/player	4 min	8	2 min	32 min

**Table 3:** Eight-week progression of SSG training

Training phase	Weeks	SSG format	Bout duration	Bouts/session	Recovery between bouts	Total active SSG time
Phase 1: Familiarization	1–2	2 vs. 2	2 min	8	2 min	16 min
Phase 2: Development I	3–4	3 vs. 3	3 min	8	2 min	24 min
Phase 3: Development II	5–6	4 vs. 4	4 min	8	2 min	32 min
Phase 4: Integration	7–8	Prescribed SSG formats according to session plan	2–4 min	8	2 min	16–32 min

**Table 4:** Structure and progression of the 8-week intervention

Phase	Weeks	SAQ group: primary emphasis	SSG group: primary emphasis	Main progression	Experimental component		Experimental component
Initial	1–2	Technique, acceleration, foot speed, and movement control	2 vs. 2	Familiarization with movement and game demands	SAQ: 12–16 min; SSG: 16 min active play		SAQ: 12–16 min; SSG: 16 min active play
Development I	3–4	Acceleration, change-of-direction speed, and multidirectional movement	3 vs. 3	Increased movement complexity and repetition volume	SAQ: 15–18 min; SSG: 24 min active play		SAQ: 15–18 min; SSG: 24 min active play
Development II	5–6	Reactive agility, acceleration, COD, and high-speed movement	4 vs. 4	Increased spatial, perceptual, and tactical demands	SAQ: 17–22 min; SSG: 32 min active play		SAQ: 17–22 min; SSG: 32 min active play
Integration	7–8	Integrated reactive SAQ, acceleration, COD, and multidirectional speed	Prescribed SSG formats according to session plan	Integration and performance maintenance	SAQ: 19–22 min; SSG: 16–32 min active play		SAQ: 19–22 min; SSG: 16–32 min active play

Both groups trained three times per week for 8 weeks, resulting in 24 sessions per group. All sessions included a 10-min standardized warm-up and a 5-min standardized cool-down

*SAQ* Speed, agility, and quickness, *SSG* Small-sided games, *COD* Change of direction

### Standardized warm-up

Each training session began with a standardized 10-min warm-up consisting of low- to moderate-intensity running, dynamic mobility exercises, movement preparation, neuromuscular activation, and progressive football-specific locomotor activities.

Participants began at relatively low intensity and progressively increased movement intensity toward moderate intensity. The same warm-up structure was used in both intervention groups to minimize differences in pre-training physiological and neuromuscular preparation.

### Speed, agility, and quickness training

The SAQ programme was designed to develop three complementary physical qualities relevant to football performance: linear acceleration and speed, multidirectional agility, and rapid movement execution or quickness.

SAQ exercises were performed immediately after the standardized warm-up. Training activities emphasized short-distance acceleration, rapid deceleration, changes of direction, multidirectional movement, rapid foot contacts, and reactive movement execution.

Exercises were performed at maximal or near-maximal movement velocity while maintaining appropriate movement technique. Participants were instructed to prioritize technical quality and movement control rather than increasing repetition volume at the expense of execution quality.

Recovery periods were incorporated between repetitions and sets to preserve movement velocity and movement quality. The training stimulus was progressively increased throughout the intervention by manipulating sets, repetitions, work duration, recovery duration, and movement complexity.

### SAQ exercise classification

The SAQ exercises were organized into three principal categories.

Speed exercises consisted primarily of short-distance acceleration drills and maximal or near-maximal acceleration over approximately 5–15 m.

Agility exercises consisted of planned acceleration–deceleration movements, shuttle-running patterns, lateral movements, and multidirectional change-of-direction tasks.

Quickness exercises included rapid-foot-contact drills, ladder-based movement patterns, short explosive movements, and reaction-based movement tasks.

Across the 8-week programme, exercise complexity and movement demands were progressively increased while maintaining the fundamental objectives of the three SAQ categories.

### SAQ coaching and training-load standardization

Coaches provided standardized technical feedback when necessary to maintain appropriate execution of the prescribed SAQ exercises. Feedback focused on acceleration mechanics, body positioning, foot placement, deceleration control, and change-of-direction technique. Coaching interventions were brief and were primarily provided before an exercise or during scheduled recovery periods to minimize disruption of the prescribed work intervals.

Participants were instructed to perform the active components at maximal or near-maximal intensity while maintaining appropriate technique. Coaches monitored compliance with the prescribed repetitions, sets, work duration, and recovery intervals throughout each session. If technical execution deteriorated substantially, the participant was instructed to prioritize movement quality rather than increase repetition speed or volume.

The work-to-recovery ratio was progressively increased in training density across the intervention, progressing from approximately 1:4 during Weeks 1–2 to approximately 1:2 during Week 8. This progression was achieved through progressive increases in work duration and/or repetition volume together with reductions in recovery duration.

### Small-sided games training

The SSG programme consisted of reduced-player-number football games designed to provide a football-specific physical stimulus while retaining the technical, tactical, perceptual, and decision-making characteristics of football.

Three SSG formats were used:

- 2 vs. 2;
- 3 vs. 3; and
- 4 vs. 4.

Pitch dimensions were scaled according to the prescribed SSG format. All games were conducted on the same playing surface using the same football size and standardized field-marking procedures.

Each SSG session consisted of eight bouts, with a fixed 2-min recovery period between consecutive bouts. Bout duration varied according to the SSG format.

The bout duration and recovery periods were controlled using a digital stopwatch. Pitch dimensions were measured and marked before each training session to ensure consistency.

### SSG training progression

The SSG intervention was progressively structured across the 8-week programme. Progression involved systematic changes in player number, playing area, bout duration, and the resulting technical, perceptual, spatial, and tactical demands.

### Standardization of SSG conditions

To ensure consistency and reproducibility throughout the intervention, all SSG sessions were conducted under standardized conditions. The same playing surface and football size were used throughout the intervention, and pitch dimensions were measured and marked before each session to ensure that the prescribed playing areas were maintained accurately.

Each SSG session consisted of eight bouts, with the duration of each bout determined by the prescribed SSG format and a fixed 2-min recovery interval between consecutive bouts. The number of players and pitch dimensions were maintained according to the prescribed experimental condition.

Teams were balanced as closely as possible according to players’ technical and tactical ability to minimize differences in competitive level between teams. No neutral or floater players were used; all participants were assigned to one of the two competing teams.

The same general playing rules were applied across training sessions, and no additional rule modifications were introduced that would systematically alter the physical or tactical demands of the games. Coaching instructions were standardized across sessions and were provided before or between bouts when required. Coaches minimized interruptions during active play to preserve continuity of play and training intensity.

Participants were instructed to remain actively involved throughout each bout and to maintain a high level of effort. The prescribed playing area per player was maintained for each SSG format at 72 m²/player in 2 vs. 2 games, 90 m²/player in 3 vs. 3 games, and 108 m²/player in 4 vs. 4 games.

### Structure and progression of the intervention

Each training session consisted exclusively of the standardized warm-up, the group-specific experimental training component, and the standardized cool-down. No additional experimental training modality was incorporated into either group.

### Cool-down

Each training session concluded with a standardized 5-min cool-down consisting of low-intensity walking or running followed by mobility and stretching exercises targeting the major lower-limb muscle groups.

The same cool-down procedure was applied to both intervention groups.

### Training adherence and monitoring

Attendance was recorded at every training session. Training adherence was calculated as:

$$\begin{aligned}&\text{Training adherence}\;(\%)\\&=\left(\text{number of sessions completed/24}\right)\\&\times100\end{aligned}$$

Any injury, illness, or other event affecting participation was documented during the intervention period.

Training sessions were supervised by qualified coaches and/or research personnel. Compliance with the prescribed sets, repetitions, work and recovery intervals, SSG bout duration, player numbers, and pitch dimensions was monitored throughout the intervention.

### Training-load monitoring

Heart rate was monitored using the Polar H10 chest-strap heart-rate sensor during the prescribed training activities according to the study protocol. The present study primarily focused on cardiovascular responses to the two active training modalities. Therefore, complementary measures of perceived exertion, GPS-derived external load, blood lactate concentration, accelerometry-derived movement load, and detailed acceleration/deceleration counts were not collected.

Consequently, the training stimulus cannot be characterized comprehensively in terms of both internal and external load. The cardiovascular findings were therefore interpreted primarily in relation to HR and blood-pressure responses rather than as a complete characterization of the physiological, metabolic, perceptual, and locomotor demands of the interventions.

### Cardiovascular outcome measures

Cardiovascular outcomes were assessed at baseline and immediately following completion of the 8-week intervention using standardized measurement procedures.

Details of the cardiovascular outcome measures, instruments, measurement procedures, and assessment timing are presented in Table 5.

The primary cardiovascular outcomes were:

1. Resting HR (beats/min);
2. Post-shuttle-run HR (beats/min);
3. SBP (mmHg); and
4. DBP (mmHg).

The principal inferential comparison was the difference in pre-to-post change between the SAQ and SSG groups.

**Table 5:** Cardiovascular outcomes and measurement procedures

Outcome	Unit	Instrument/procedure	Assessment time
Resting HR	beats/min	Polar H10 chest-strap heart-rate sensor following 10-min seated rest	Pre and post
Post-shuttle-run HR	beats/min	Polar H10 chest-strap heart-rate sensor recorded immediately after termination of the 20-m shuttle-run test	Pre and post
SBP	mmHg	Omron HEM-7120 automated upper-arm oscillometric monitor; mean of three readings obtained after 10-min seated rest	Pre and post
DBP	mmHg	Omron HEM-7120 automated upper-arm oscillometric monitor; mean of three readings obtained after 10-min seated rest	Pre and post

*Abbreviations*: *HR* Heart rate, *SBP* Systolic blood pressure, *DBP* Diastolic blood pressure

### Resting heart rate

Participants rested quietly in a seated position for 10 min before resting HR measurement. During the rest period, participants were instructed to remain relaxed, avoid talking, and minimize unnecessary movement.

Heart rate was measured using a Polar H10 chest-strap heart-rate sensor (Polar Electro Oy, Kempele, Finland), positioned around the participant’s chest according to the manufacturer’s instructions.

The HR recorded following the standardized 10-min seated rest period was used as the resting HR outcome. The same device and measurement procedure were used at baseline and post-intervention.

### 20-m shuttle run test

Cardiorespiratory fitness was assessed using the 20-m multistage shuttle-run test (20-mSRT), also known as the beep test, according to the protocol originally described by Léger et al., [28]. The test was conducted outdoors on a level artificial-turf soccer field. Two parallel lines were marked 20 m apart, and participants ran continuously between the lines in synchronization with the auditory pacing signals.

The pacing audio used during the test was the same recording for all participants and assessment sessions. The audio was obtained from the YouTube recording “MULTI STAGE FITNESS TEST (20 M BLEEP TEST) AUDIO” by Luke Leon Brimble Fitness and was used consistently during both baseline and post-intervention assessments. The test began at a running speed of 8.5 km·h⁻¹, with the running speed progressively increasing by 0.5 km·h⁻¹ at each stage, consistent with the Léger protocol. Participants received standardized verbal encouragement and were instructed to maintain the required pace for as long as possible.

The test was terminated when a participant failed to reach the designated 20-m line in time with the auditory signal on two consecutive occasions despite verbal encouragement, or voluntarily stopped because of exhaustion and was unable to continue. The final successfully completed stage was recorded as the test score.

Heart rate was continuously monitored using a Polar H10 chest-strap heart-rate sensor. The HR recorded immediately after test termination was used as the post-shuttle-run HR outcome. The same 20-m distance, artificial-turf surface, audio recording, equipment, instructions, and testing procedures were used during both baseline and post-intervention assessments.

### Test termination criteria

The test was terminated when a participant voluntarily stopped because of fatigue and was unable to continue, or when the participant failed to reach the designated 20-m line in synchronization with the auditory signal on two consecutive occasions despite verbal encouragement.

The final successfully completed stage was recorded as the test score.

The same 20-m distance, artificial-turf surface, instructions, equipment, and audio recording were used at baseline and post-intervention.

### Heart-rate response to the 20-m shuttle-run test

Heart rate was continuously monitored throughout the 20-m shuttle-run test using the Polar H10 chest-strap heart-rate sensor.

The HR recorded immediately after termination of the 20-m shuttle-run test was used as the post-shuttle-run HR outcome.

The same heart-rate sensor and recording procedure were used at both assessment points.

### Blood pressure

Blood pressure was assessed following a standardized 10-min seated rest period using an automated upper-arm oscillometric blood-pressure monitor (Omron HEM-7120, Omron Healthcare Co., Ltd.). The HEM-7120 operates using the oscillometric method.

Participants were seated with their back supported, feet flat on the floor, legs uncrossed, and the measurement arm supported at approximately heart level. Participants were instructed to remain quiet and relaxed throughout the measurement procedure.

The standard HEM-7120 medium arm cuff is designed for an upper-arm circumference of 22–32 cm; the manufacturer also documents an optional large cuff for 32–42 cm.

Three consecutive BP measurements were obtained at 1-min intervals following the 10-min seated rest period. The mean of the three readings was calculated separately for SBP and DBP and used for statistical analysis.

The same device, cuff, participant positioning, rest period, measurement interval, and averaging procedure were used at baseline and post-intervention.

### Standardization of cardiovascular testing

To minimize measurement-related variation, baseline and post-intervention assessments were conducted using the same general procedures, equipment, testing environment, and assessment sequence.

Participants were instructed to attend testing in a rested condition and to avoid strenuous physical activity immediately before assessment.

Upon arrival, participants underwent a standardized 10-min seated rest period before resting cardiovascular measurements were obtained.

Resting HR was then recorded using the Polar H10 heart-rate sensor. Blood pressure was subsequently measured using the Omron HEM-7120 following the standardized three-reading procedure, with measurements obtained at 1-min intervals and the mean value used for analysis.

The 20-m shuttle-run test was subsequently conducted using the standardized 20-m distance and audio pacing protocol. Heart rate was continuously monitored during the test, and the HR recorded immediately after test termination was used as the post-shuttle-run HR outcome.

The same Polar H10 sensor, Omron HEM-7120 monitor, cuff, 20-m testing distance, artificial-turf surface, test instructions, and shuttle-run audio recording were used at both assessment points.

### Materials and measurement equipment

Heart rate was measured using a Polar H10 chest-strap heart-rate sensor (Polar Electro Oy, Kempele, Finland). The sensor was used for both resting HR and continuous monitoring during the 20-m shuttle-run test.

Blood pressure was measured using an Omron HEM-7120 automated upper-arm oscillometric blood-pressure monitor (Omron Healthcare Co., Ltd.). The standard medium cuff covers an upper-arm circumference of 22–32 cm; an optional large cuff is available for 32–42 cm.

A digital stopwatch was used to control SSG bout and recovery durations, and standardized field-marking equipment was used to establish the prescribed SSG pitch dimensions.

### Statistical analysis

All statistical analyses were performed using IBM SPSS Statistics version 26.0 (IBM Corp., Armonk, NY, USA). Continuous variables are presented as mean ± standard deviation (SD). The distributional characteristics of continuous variables were examined before inferential analyses.

Baseline demographic, anthropometric, training, and cardiovascular characteristics were compared between the SAQ and SSG groups using independent-samples *t* tests.

Changes in cardiovascular outcomes from pre- to post-intervention were examined using a two-way mixed-design analysis of variance (ANOVA), with time (pre-intervention vs. post-intervention) as the within-subject factor and training group (SAQ vs. SSG) as the between-subject factor. The primary inferential effect was the time × group interaction, which assessed whether the pre-to-post change in each cardiovascular outcome differed between the two training groups.

For each cardiovascular outcome, the pre-to-post change was calculated as the post-intervention value minus the pre-intervention value. The between-group difference in change was calculated as the SAQ change minus the SSG change. The 95% confidence interval (CI) for each between-group difference in change was reported to quantify the precision of the estimated difference. Partial eta squared (ηp²) was reported as the effect size for the time × group interaction, whereas Cohen’s *d* was used to describe the standardized within-group pre-to-post change. Effect sizes were interpreted together with the direction and magnitude of the observed changes and the 95% CI of the between-group differences.

The four cardiovascular outcomes were analyzed separately as prespecified outcome measures using the same mixed-design ANOVA framework. No formal adjustment for multiple comparisons was applied across the four cardiovascular outcomes. Accordingly, the statistical findings were interpreted with consideration of the potential inflation of the family-wise Type I error rate. The time × group interaction was considered the primary inferential test for each cardiovascular outcome, and no additional post-hoc tests were performed.

Statistical significance was set at *P* < 0.05. Because baseline differences were observed for resting heart rate, systolic blood pressure, and diastolic blood pressure, between-group differences in pre-to-post trajectories for these outcomes were interpreted cautiously. Significant time × group interactions were interpreted as evidence that the patterns of change differed between the SAQ and SSG groups rather than as definitive evidence that one training modality produced greater cardiovascular adaptation.

## Results

### Participant characteristics

Thirty-six male youth football players participated in the study, with 18 participants in the Speed, Agility, and Quickness (SAQ) group and 18 in the Small-Sided Games (SSG) group. All participants completed the 8-week intervention and the pre- and post-intervention assessments. Baseline demographic, anthropometric, training, and cardiovascular characteristics are presented in Table 6. No statistically significant between-group differences were observed for age, height, body mass, training age, or post-shuttle-run heart rate (*P* > 0.05). Significant between-group differences were observed for resting heart rate, resting systolic blood pressure, and resting diastolic blood pressure at baseline (all *P* < 0.001).

**Table 6:** Baseline characteristics of participants

Variable	SAQ group (*n* = 18)	SSG group (*n* = 18)	*P* value
Age (years)	15.08 ± 0.74	15.12 ± 0.69	0.868
Height (cm)	167.4 ± 5.8	168.1 ± 6.1	0.726
Body mass (kg)	61.7 ± 6.4	62.3 ± 5.9	0.772
Training age (years)	4.3 ± 1.1	4.5 ± 1.0	0.572
Resting HR (beats/min)	72.20 ± 1.77	74.66 ± 0.48	< 0.001
Post-shuttle-run HR (beats/min)	163.50 ± 2.86	164.66 ± 2.27	0.187
Resting SBP (mmHg)	118.27 ± 1.59	123.46 ± 2.29	< 0.001
Resting DBP (mmHg)	73.77 ± 1.06	80.66 ± 0.97	< 0.001

Data are presented as mean ± standard deviation (SD)

*Abbreviations*: *SAQ* Speed, Agility, and Quickness, *SSG* Small-sided games, *HR* Heart rate, *SBP* Systolic blood pressure, *DBP* Diastolic blood pressure

### Cardiovascular outcomes

**Fig. 2:**
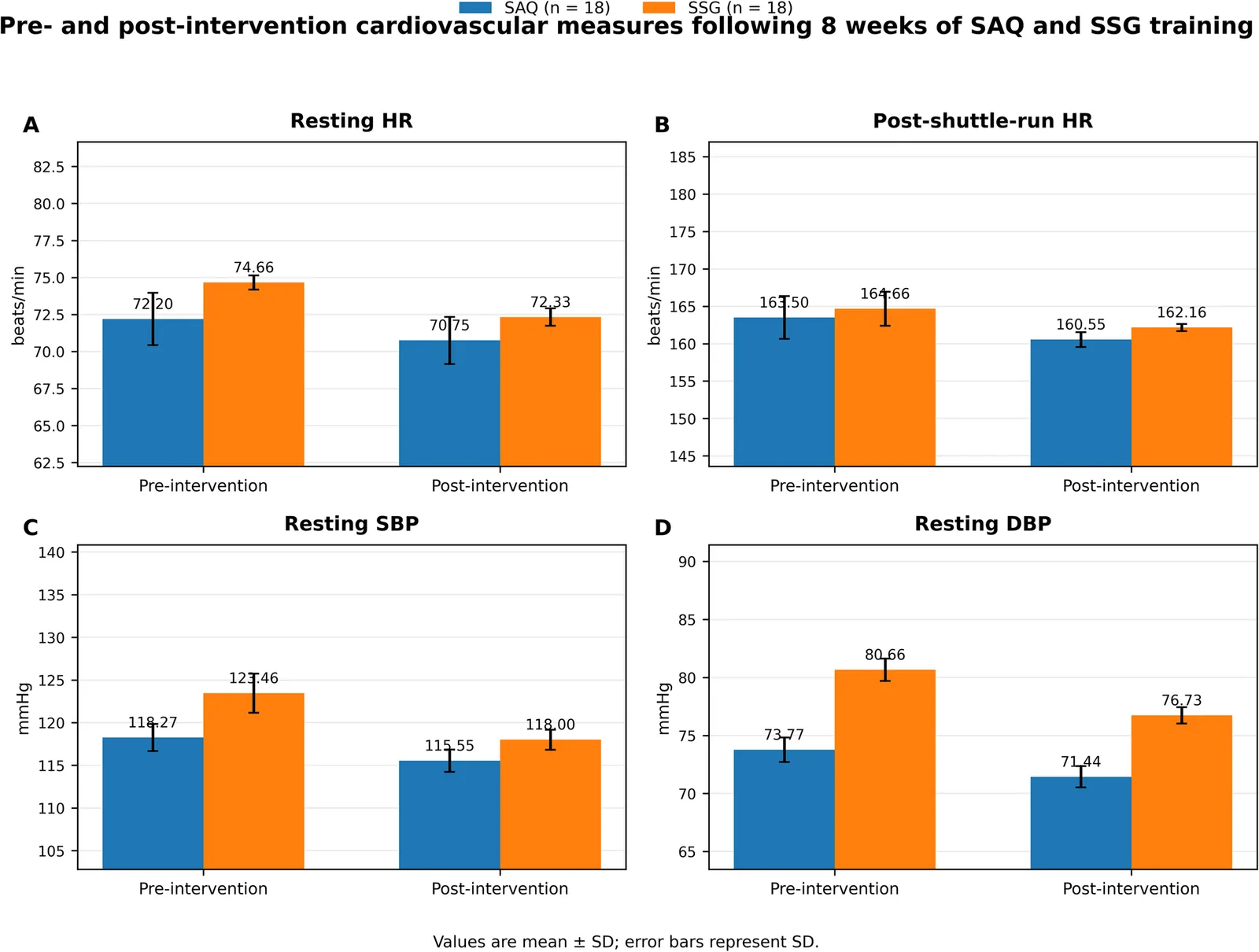
Pre- and post-intervention cardiovascular measures following 8 weeks of SAQ and SSG training. **A** Resting heart rate; (**B**) post-shuttle-run heart rate; (**C**) resting systolic blood pressure; and (**D**) resting diastolic blood pressure. Data are presented as mean ± SD. SAQ, speed, agility, and quickness; SSG, small-sided games; HR, heart rate; SBP, systolic blood pressure; DBP, diastolic blood pressure.

The cardiovascular measures before and after the 8-week intervention are presented in Table 7 and illustrated in Fig. 2. Both training groups showed reductions in resting heart rate, post-shuttle-run heart rate, systolic blood pressure, and diastolic blood pressure following the intervention.

**Table 7:** Cardiovascular responses before and after the 8-week intervention

Outcome	SAQ Pre, mean ± SD	SAQ Post, mean ± SD	SSG Pre, mean ± SD	SSG Post, mean ± SD
Resting HR (beats/min)	72.20 ± 1.77	70.75 ± 1.59	74.66 ± 0.48	72.33 ± 0.59
Post-shuttle-run HR (beats/min)	163.50 ± 2.86	160.55 ± 0.99	164.66 ± 2.27	162.16 ± 0.48
SBP (mmHg)	118.27 ± 1.59	115.55 ± 1.31	123.46 ± 2.29	118.00 ± 1.17
DBP (mmHg)	73.77 ± 1.06	71.44 ± 0.92	80.66 ± 0.97	76.73 ± 0.70

*Abbreviations*: *SAQ* Speed, Agility, and Quickness, *SSG* Small-sided games, *HR* Heart rate, *SBP* Systolic blood pressure, *DBP* Diastolic blood pressure, *SD* Standard deviation

Resting heart rate decreased from 72.20 ± 1.77 to 70.75 ± 1.59 beats/min in the SAQ group and from 74.66 ± 0.48 to 72.33 ± 0.59 beats/min in the SSG group. Post-shuttle-run heart rate decreased from 163.50 ± 2.86 to 160.55 ± 0.99 beats/min in the SAQ group and from 164.66 ± 2.27 to 162.16 ± 0.48 beats/min in the SSG group. Systolic blood pressure decreased from 118.27 ± 1.59 to 115.55 ± 1.31 mmHg in the SAQ group and from 123.46 ± 2.29 to 118.00 ± 1.17 mmHg in the SSG group. Diastolic blood pressure decreased from 73.77 ± 1.06 to 71.44 ± 0.92 mmHg in the SAQ group and from 80.66 ± 0.97 to 76.73 ± 0.70 mmHg in the SSG group (Fig. 2).

### Pre-to-post changes and between-group differences

The pre-to-post changes, between-group differences in change, confidence intervals, and effect sizes are presented in Table 8.

**Table 8:** Pre-to-post changes and effect sizes

Outcome	SAQ change	SSG change	Difference in change (SAQ − SSG)	95% CI	SAQ Cohen’s d	SSG Cohen’s d	Time × Group ηp²
Resting HR (beats/min)	−1.45	−2.33	+ 0.88	0.32 to 1.44	0.20	1.02	0.24
Post-shuttle-run HR (beats/min)	−2.95	−2.50	−0.45	−0.66 to − 0.24	0.32	0.36	0.38
SBP (mmHg)	−2.72	−5.46	+ 2.74	1.58 to 3.90	0.44	0.71	0.42
DBP (mmHg)	−2.33	−3.93	+ 1.60	1.14 to 2.06	0.55	1.09	0.61

Change scores were calculated as post-intervention values minus pre-intervention values. The difference in change represents the SAQ change minus the SSG change. The 95% CI represents the confidence interval for the between-group difference in pre-to-post change. Cohen’s *d* represents the standardized within-group pre-to-post effect size, whereas partial eta squared (ηp²) represents the effect size of the time × group interaction.

*HR* Heart rate, *SBP* Systolic blood pressure, *DBP* Diastolic blood pressure

Resting heart rate decreased by 1.45 beats/min in the SAQ group and by 2.33 beats/min in the SSG group. The between-group difference in pre-to-post change was 0.88 beats/min (SAQ − SSG; 95% CI, 0.32 to 1.44), indicating a greater numerical reduction in resting heart rate in the SSG group.

Post-shuttle-run heart rate decreased by 2.95 beats/min in the SAQ group and by 2.50 beats/min in the SSG group. The between-group difference in change was − 0.45 beats/min (95% CI, − 0.66 to − 0.24), indicating a slightly greater numerical reduction in post-shuttle-run heart rate in the SAQ group.

Systolic blood pressure decreased by 2.72 mmHg in the SAQ group and by 5.46 mmHg in the SSG group. The between-group difference in change was 2.74 mmHg (95% CI, 1.58 to 3.90), indicating a greater numerical reduction in systolic blood pressure in the SSG group.

Diastolic blood pressure decreased by 2.33 mmHg in the SAQ group and by 3.93 mmHg in the SSG group. The between-group difference in change was 1.60 mmHg (95% CI, 1.14 to 2.06), indicating a greater numerical reduction in diastolic blood pressure in the SSG group.

### Between-group effects

The repeated-measures analysis demonstrated significant time × group interactions for all four cardiovascular outcomes. For resting heart rate, the time × group interaction was significant, *F*(1,32) = 10.11, *P* = 0.003, ηp² = 0.24, with a between-group difference in pre-to-post change of 0.88 beats/min (95% CI, 0.32 to 1.44). The corresponding within-group Cohen’s *d* values were 0.20 for the SAQ group and 1.02 for the SSG group.

For post-shuttle-run heart rate, the time × group interaction was significant, *F*(1,32) = 19.61, *P* < 0.001, ηp² = 0.38, with a between-group difference in pre-to-post change of − 0.45 beats/min (95% CI, − 0.66 to − 0.24). The corresponding within-group Cohen’s *d* values were 0.32 for the SAQ group and 0.36 for the SSG group.

For systolic blood pressure, a significant time × group interaction was observed, *F*(1,32) = 23.17, *P* < 0.001, ηp² = 0.42, with a between-group difference in pre-to-post change of 2.74 mmHg (95% CI, 1.58 to 3.90). The corresponding within-group Cohen’s *d* values were 0.44 for the SAQ group and 0.71 for the SSG group.

For diastolic blood pressure, the time × group interaction was also significant, *F*(1,32) = 50.05, *P* < 0.001, ηp² = 0.61, with a between-group difference in pre-to-post change of 1.60 mmHg (95% CI, 1.14 to 2.06). The corresponding within-group Cohen’s *d* values were 0.55 for the SAQ group and 1.09 for the SSG group.

The direction of the between-group differences indicated greater numerical reductions in resting heart rate, systolic blood pressure, and diastolic blood pressure in the SSG group, whereas the SAQ group showed a slightly greater numerical reduction in post-shuttle-run heart rate. The interaction effect sizes ranged from ηp² = 0.24 to 0.61, with the largest effect observed for diastolic blood pressure. These findings indicate that the two training groups exhibited different pre-to-post cardiovascular response trajectories over the 8-week intervention. The corresponding mean changes, between-group differences, 95% CIs, and effect sizes are presented in Table 8.

## Discussion

The present study compared cardiovascular responses to an 8-week Speed, Agility, and Quickness (SAQ) programme and small-sided games (SSGs) in male youth football players. Both training approaches were associated with reductions in resting heart rate (HR), post-shuttle-run HR, systolic blood pressure (SBP), and diastolic blood pressure (DBP). Significant time × group interactions were observed for all four outcomes, with partial eta-squared values ranging from 0.24 to 0.61. The direction and magnitude of change, however, differed between outcomes. SSG training produced larger numerical reductions in resting HR, SBP, and DBP, whereas SAQ training produced a slightly greater reduction in post-shuttle-run HR. Taken together, these findings suggest that the two training approaches were associated with distinct cardiovascular response patterns following repeated training exposure.

### Cardiovascular responses following SAQ and SSG training

Resting HR decreased by 1.45 beats/min in the SAQ group and by 2.33 beats/min in the SSG group. Resting HR is influenced by autonomic regulation, stroke volume, aerobic fitness, training status, and recovery and can therefore provide a useful indicator of cardiovascular response when considered alongside other physiological measures [26, 27]. Although the absolute changes observed in the present study were modest, the significant time × group interaction (ηp² = 0.24) indicates that the two groups followed different pre-to-post trajectories.

The greater numerical reduction in resting HR in the SSG group may be related to the intermittent and multidirectional nature of game-based training. During SSGs, players repeatedly accelerate, decelerate, change direction, reposition, and perform football-specific actions in response to changing game situations [7, 11, 29]. These characteristics can produce substantial cardiovascular loading, although the physiological demands of SSGs are highly dependent on their design. Player number, pitch dimensions, rules, tactical organization, and team strategy can substantially influence HR and movement demands [10, 12, 14–20]. Therefore, the present findings should be interpreted in relation to the specific SSG format used rather than generalized to SSG training as a whole.

A similar pattern was observed for blood pressure. SBP decreased by 2.72 mmHg in the SAQ group and 5.46 mmHg in the SSG group, while DBP decreased by 2.33 and 3.93 mmHg, respectively. The time × group effects were ηp² = 0.42 for SBP and ηp² = 0.61 for DBP, with the largest interaction observed for DBP. These results indicate clear differences in the temporal response patterns between the training conditions. Nevertheless, the underlying physiological mechanisms cannot be established because variables such as autonomic activity, cardiac output, vascular resistance, endothelial function, and arterial stiffness were not directly assessed. The findings should therefore be interpreted as changes in measured cardiovascular variables rather than evidence of specific cardiac or vascular adaptations.

The larger reductions in SBP and DBP following SSG training may reflect the repeated combination of intermittent running and changes in exercise intensity characteristic of football-specific game play [7, 13, 21]. Previous research has shown that SSGs can provide an effective conditioning stimulus for endurance and physical-performance development [23–25, 30]. The present results extend this literature by showing that an 8-week SSG programme was associated with changes in resting cardiovascular measures. However, because baseline differences were present for several resting variables, these findings should not be interpreted as definitive evidence that SSG training produces greater cardiovascular adaptation than SAQ training.

### Post-shuttle-run heart-rate response

The post-shuttle-run HR response differed from the resting measures. HR following the shuttle-run test decreased by 2.95 beats/min in the SAQ group and by 2.50 beats/min in the SSG group, with a significant time × group interaction (ηp² = 0.38). Thus, the response to a standardized exercise challenge did not follow exactly the same pattern as the resting cardiovascular measures.

One possible explanation is the specific nature of the SAQ stimulus. SAQ training emphasizes acceleration, rapid changes of direction, explosive movement, and neuromuscular coordination. Although it is not primarily an endurance-training modality, repeated high-intensity efforts can impose substantial cardiovascular demands, particularly when bouts are performed repeatedly with limited recovery [1, 4, 6, 8, 31]. Previous studies have reported improvements in sprinting, agility, change-of-direction ability, and related physical-performance characteristics following SAQ-oriented training in young football players [6, 8, 9, 22].

The slightly greater reduction in post-shuttle-run HR in the SAQ group may therefore reflect the specific demands imposed by repeated high-intensity movement. This finding should not, however, be interpreted as evidence of greater overall cardiovascular effectiveness. Rather, it highlights that cardiovascular responses may depend on whether training emphasizes repeated explosive actions and neuromuscular demands or the more integrated physical and football-specific demands of SSGs.

### Training-specific physiological demands

SAQ training and SSGs provide different training stimuli and consequently should not be regarded as interchangeable interventions. SAQ training allows coaches to manipulate acceleration, agility, rapid changes of direction, and high-intensity movement in a relatively controlled environment [6, 8, 9, 22]. SSGs expose players to a more variable combination of locomotor activity, technical execution, tactical positioning, perception, and decision-making [29, 32, 33].

The additional football-specific demands of SSGs may influence movement intensity and distribution throughout a training session. Players must continuously respond to teammates, opponents, space, and task constraints, which can alter running patterns and recovery periods. Nevertheless, the present study did not directly assess technical performance, tactical behavior, cognitive load, perceptual demands, or decision-making. These factors should therefore be considered plausible characteristics of the training stimulus rather than mechanisms demonstrated by the present data.

This distinction also helps explain why a larger reduction in a particular cardiovascular variable should not be equated with greater overall training effectiveness. Youth football performance depends on multiple interacting physical and football-specific qualities. A training method may be advantageous for one performance objective while providing a different stimulus for another. Accordingly, the present findings are more appropriately interpreted in terms of training-specific response patterns than as evidence supporting a universal hierarchy between SAQ and SSG training.

### Comparison with previous SSG research

The present findings are consistent with previous research demonstrating that SSGs can impose substantial cardiovascular and physical demands on football players. Acute physiological responses during SSGs are influenced by player number, pitch dimensions, rules, tactical organization, and other contextual factors [10, 14–20, 34]. For example, changes in relative playing area can alter running and physiological demands, while rule modifications can affect the intensity and distribution of activity [13, 20, 34]. Consequently, SSGs should be considered adaptable training environments rather than a single standardized training modality.

The current study differs from much of the existing SSG literature because it examined changes in cardiovascular measures across an 8-week intervention rather than the acute physiological response to individual SSG sessions. Previous studies and reviews have shown that SSGs can provide effective conditioning stimuli and can produce improvements in endurance and physical performance [23–25, 30, 35]. Moran et al. [23], for example, reported beneficial effects of SSG-based training on endurance-related outcomes in male youth football players, while Kunz et al. [24] and Clemente et al. [25] reported meaningful physiological and physical-performance responses associated with SSG training. Pop et al. [35] additionally reported physical and motivational responses following SSG-based interventions in youth soccer players.

Direct comparison should nevertheless be made cautiously. An elevated HR during an individual SSG reflects the immediate physiological demand of that session, whereas the present study evaluated changes after repeated exposure over 8 weeks. Acute exercise intensity and longer-term adaptation are related but distinct concepts [7, 23, 24, 30]. The current findings therefore contribute to the literature by examining cardiovascular response trajectories following a sustained training intervention rather than by estimating the acute intensity of SSG exercise.

### Magnitude and practical interpretation of the effects

The interaction effect sizes provide useful information beyond statistical significance. Partial eta-squared values ranged from 0.24 for resting HR to 0.61 for DBP, indicating substantial differences between the groups in their pre-to-post response patterns. The between-group differences in change were 0.88 beats/min for resting HR (95% CI, 0.32 to 1.44), − 0.45 beats/min for post-shuttle-run HR (95% CI, − 0.66 to − 0.24), 2.74 mmHg for SBP (95% CI, 1.58 to 3.90), and 1.60 mmHg for DBP (95% CI, 1.14 to 2.06). These confidence intervals provide an indication of the precision of the estimated differences between the two training groups.

The direction of these differences indicates that the SSG group demonstrated greater reductions in resting HR, SBP, and DBP, whereas the SAQ group demonstrated a slightly greater reduction in post-shuttle-run HR. Although the confidence intervals for all four between-group differences did not include zero, the magnitude of the observed differences should be interpreted in relation to the baseline characteristics of the groups and the overall study design. In particular, the baseline differences observed for resting HR, SBP, and DBP may have contributed to the different pre-to-post trajectories.

The interaction effect sizes were ηp² = 0.24 for resting HR, ηp² = 0.38 for post-shuttle-run HR, ηp² = 0.42 for SBP, and ηp² = 0.61 for DBP. The largest interaction effect was observed for DBP, followed by SBP, post-shuttle-run HR, and resting HR. However, these effect sizes should not be interpreted independently of the absolute magnitude of change or the baseline imbalance between groups. The results therefore provide evidence of different cardiovascular response patterns under the conditions investigated rather than definitive evidence of clinically superior cardiovascular adaptation with either training modality.

Because the four cardiovascular outcomes were analyzed separately without a formal adjustment for multiple comparisons, the statistical findings should also be interpreted with consideration of the potential inflation of the family-wise Type I error rate.

From an applied perspective, the results suggest that training selection should be guided primarily by the objectives of the training session. SSGs may be particularly useful when coaches seek to combine cardiovascular conditioning with technical and tactical activity, whereas SAQ training may be more appropriate when the emphasis is on acceleration, agility, rapid changes of direction, and neuromuscular qualities [6, 8, 9, 22]. These approaches can therefore serve different functions within a broader youth football training programme.

### Baseline differences and interpretation of the findings

An important limitation in interpreting the between-group effects is that the groups differed at baseline for resting HR, SBP, and DBP, whereas post-shuttle-run HR did not differ significantly at baseline. The SSG group therefore entered the intervention with higher resting cardiovascular values.

This imbalance is relevant because a greater numerical reduction in a variable can partly reflect differences in starting values. Although the significant time × group interactions demonstrate that the groups changed differently over time, they do not establish that the training modality alone caused the observed differences. This issue is particularly important for resting HR, SBP, and DBP, for which baseline differences were evident.

The findings should consequently be interpreted as differences in pre-to-post response trajectories rather than definitive estimates of the causal superiority of one training modality. Future randomized studies should use baseline-adjusted analyses, such as ANCOVA or mixed-effects models incorporating baseline values, particularly when meaningful baseline imbalance is present.

### Youth football considerations

The developmental characteristics of youth players provide additional context for interpreting the findings, as individual differences in maturation, body size, fitness level, and training history may contribute to variability in cardiovascular and physical responses. Previous research has also demonstrated considerable variability in the physical and physiological demands experienced by young football players during SSGs [10, 15, 16, 21].

The present results therefore support an individualized approach to training prescription. SSGs offer the advantage of combining conditioning with football-specific technical and tactical activity [29, 32, 33], whereas SAQ training permits more precise manipulation of acceleration, agility, and rapid movement demands [6, 8, 9, 22]. Rather than treating the two approaches as competing alternatives, coaches may use them according to the developmental and performance objectives of individual sessions.

### Practical implications

The practical relevance of these findings lies primarily in the different training characteristics of the two approaches. SSGs can simultaneously expose players to cardiovascular loading and technical, tactical, perceptual, and decision-making demands [29, 32–34]. Their intensity can be modified through pitch dimensions, player number, task rules, work-to-rest structure, and tactical constraints [7, 13–18, 20]. Such flexibility allows coaches to manipulate the overall training stimulus while retaining football-specific characteristics.

SAQ training provides greater control over acceleration, agility, rapid changes of direction, and repeated high-intensity actions. Previous research supports its use for developing several physical-performance characteristics in young football players [6, 8, 9, 22]. The present findings further indicate that SAQ training can be accompanied by changes in cardiovascular response, despite its primary emphasis on high-intensity neuromuscular actions.

The two approaches can therefore be integrated within the same training programme. Coaches may use SSGs when the objective is to develop football-specific conditioning alongside technical and tactical qualities, while SAQ exercises can be incorporated when more targeted development of speed-related and neuromuscular qualities is required. The appropriate combination will depend on training objectives, player characteristics, stage of development, and the broader periodized programme.

### Strengths and limitations

This study has several strengths. It directly compared two commonly used football-conditioning approaches within the same experimental framework and examined responses following an 8-week intervention rather than relying solely on acute exercise responses. The inclusion of both resting and post-shuttle-run HR provided complementary information regarding cardiovascular responses at rest and following a standardized exercise challenge. In addition, reporting interaction effect sizes together with absolute changes facilitates interpretation of the magnitude and direction of the observed differences.

Several limitations should also be considered. First, the sample was relatively small and consisted exclusively of male youth football players, which limits generalizability to female players, other age groups, and different competitive levels. Second, only HR and blood-pressure measures were assessed; therefore, the study cannot establish the physiological mechanisms underlying the observed changes or determine whether they represent comprehensive improvements in cardiovascular fitness. Measures such as autonomic regulation, cardiac output, endothelial function, and vascular stiffness were not included.

Third, baseline differences in resting HR, SBP, and DBP complicate the interpretation of the between-group effects. Although participants were randomly allocated, the groups were not fully comparable at baseline for these variables. The observed differences should therefore be interpreted cautiously and not as definitive evidence of superior cardiovascular adaptation following SSG training.

Fourth, although random allocation was reported, the sequence-generation and allocation-concealment procedures were not documented in sufficient detail to permit full evaluation of the allocation process. Formal blinding of outcome assessors was also not confirmed. These issues may introduce potential selection or assessment bias and should be addressed through transparent randomization procedures and assessor blinding in future trials. Nevertheless, the cardiovascular outcomes were objective physiological measures obtained using standardized equipment and testing procedures.

Fifth, the study did not include continuous monitoring of training load or complementary measures such as GPS-derived external load, rating of perceived exertion, blood lactate, or technical and tactical performance. Consequently, the relationship between the actual training stimulus and the observed cardiovascular changes cannot be determined directly. Future studies should combine cardiovascular, internal-load, external-load, technical, tactical, perceptual, cognitive, and neuromuscular measures to provide a more comprehensive assessment of training adaptation.

Finally, the physiological demands of SSGs depend strongly on their specific configuration, including pitch dimensions, player number, rules, tactical strategy, and individual player characteristics [14–20, 34]. The findings should therefore not be generalized to all SSG formats without considering these contextual factors.

### Future research

Future research should adopt larger randomized samples and incorporate baseline-adjusted statistical approaches to improve estimation of between-group effects. Studies should also account for biological maturation, baseline fitness, training history, and habitual activity when examining responses in youth players.

A multidimensional assessment framework would provide further insight by combining HR and blood pressure with GPS-derived external load, perceived exertion, blood lactate, and longitudinal fitness measures. Concurrent assessment of technical execution, tactical behavior, decision-making, perceptual demands, and neuromuscular performance would also clarify whether the broader characteristics of SSGs contribute to their distinct response profile.

Longer interventions would be particularly valuable for determining whether the cardiovascular response patterns observed after 8 weeks persist and whether they are accompanied by changes in aerobic fitness and football performance. Examining individual response trajectories would additionally help determine whether player-specific characteristics influence adaptation to SAQ and SSG training and could support more individualized training prescription.

## Conclusions

Both SAQ and SSG training were associated with reductions in resting HR, post-shuttle-run HR, SBP, and DBP following the 8-week intervention, with significant differences in pre-to-post response trajectories between the groups. SSG training showed larger numerical reductions in resting HR, SBP, and DBP, whereas SAQ training showed a slightly greater reduction in post-shuttle-run HR. The findings therefore indicate different cardiovascular response patterns rather than establishing a universal advantage of either training approach.

Because baseline differences were present for resting HR, SBP, and DBP, the between-group findings should be interpreted cautiously. The present data do not provide sufficient evidence to conclude that SSG training produces superior long-term cardiovascular adaptation. Rather, the results suggest that SAQ and SSG training can provide distinct physiological stimuli within youth football conditioning.

From a practical perspective, the two methods may be viewed as complementary. SSGs allow cardiovascular conditioning to be integrated with technical, tactical, perceptual, and decision-making demands, whereas SAQ training provides a more targeted stimulus for acceleration, agility, rapid changes of direction, and neuromuscular performance. Coaches should therefore select and combine these modalities according to the objectives of individual training sessions and the broader developmental needs of youth football players.

## Supplementary Information

Supplementary Material 1.

## Data Availability

All raw data underlying the findings of this study are available from the corresponding author upon reasonable request and without undue restriction.
