# Supplementary material for: Comparative changes in cardiovascular measures following speed, agility, and quickness training and small-sided games in youth football players: a randomized trial
Source: BMC Sports Sci Med Rehabil. 2026 Sep 10;18:419. doi: 10.1186/s13102-026-02048-2 (PMC13576338; doi:10.1186/s13102-026-02048-2)
Supplement: Supplementary file 1 [file 13102_2026_2048_MOESM1_ESM.docx]

# Completed CONSORT Checklist for the Submitted Randomized Controlled Trial

Comparative Effects of Speed, Agility, and Quickness Training and Small-Sided Games on Cardiovascular Response in Youth Football Players: A Randomized Controlled Trial

| Section/Item | CONSORT Item | Reported | Manuscript Location |
| --- | --- | --- | --- |
| Title/Abstract 1a | Randomised trial identified in title | Yes | Title page |
| Title/Abstract 1b | Structured abstract | Yes | Abstract |
| Introduction 2a | Scientific background and rationale | Yes | Introduction |
| Introduction 2b | Specific objectives/hypotheses | Yes | End of Introduction |
| Methods 3a | Trial design | Yes | Study Design |
| Methods 3b | Changes to methods after commencement | No changes | Study Design |
| Methods 4a | Eligibility criteria | Yes | Participants |
| Methods 4b | Settings and locations | Yes | Participants |
| Methods 5 | Interventions | Yes | Intervention Protocols |
| Methods 6a | Outcome measures | Yes | Physiological Measurements |
| Methods 6b | Changes to outcomes | No | Not applicable |
| Methods 7a | Sample size determination | Yes | Sample Size Calculation |
| Methods 7b | Interim analyses/stopping guidelines | Not applicable | Study Design |
| Methods 8a | Sequence generation | Yes | Randomization and Group Allocation |
| Methods 8b | Type of randomization | Yes | Randomization and Group Allocation |
| Methods 9 | Allocation concealment | Yes | Sealed opaque envelopes |
| Methods 10 | Implementation | Partially | Randomization section |
| Methods 11a | Blinding | Not reported | Should be added |
| Methods 11b | Similarity of interventions | Not applicable | Interventions differ by design |
| Methods 12a | Statistical methods | Yes | Statistical Analysis |
| Methods 12b | Additional analyses | Yes | ANOVA and effect sizes |
| Results 13a | Participant flow | Yes | Figure 1 |
| Results 13b | Losses/exclusions | Yes, none | Figure 1 |
| Results 14a | Recruitment dates | Partially | 8-week study reported |
| Results 14b | Why trial ended | Completed as planned | Study Design |
| Results 15 | Baseline data | Yes | Table 2 |
| Results 16 | Numbers analyzed | Yes | 36 analyzed |
| Results 17a | Outcome results with effect size | Yes | Tables 3–5 |
| Results 17b | Binary outcomes | Not applicable | No binary outcomes |
| Results 18 | Ancillary analyses | Yes | ANOVA interaction analyses |
| Results 19 | Harms | Yes, none reported | Study Design/Results |
| Discussion 20 | Limitations | Yes | Discussion |
| Discussion 21 | Generalisability | Yes | Discussion |
| Discussion 22 | Interpretation | Yes | Discussion and Conclusion |
| Other 23 | Registration | Yes | Abstract, NCT07633210 |
| Other 24 | Protocol access | Not reported | Should be added if available |
| Other 25 | Funding | Yes | Funding statement |
